# Factors Influencing Adherence to Pediatric Antiretroviral Therapy: Experience from a Tertiary Care Center in Oman

**DOI:** 10.7759/cureus.106842

**Published:** 2026-04-11

**Authors:** Zulfa Al Shibli, Basma Al Jabri, Jalila Al Namani, Hilal AL Hashami

**Affiliations:** 1 Child Health, Royal Hospital, Muscat, OMN; 2 Pharmaceutical Care Department, Royal Hospital, Muscat, OMN; 3 Nursing, Royal Hospital, Muscat, OMN

**Keywords:** adherence, antiretroviral therapy, children, hiv, oman

## Abstract

Introduction

Antiretroviral therapy (ART) compliance is essential for successful treatment outcomes of children living with human immunodeficiency virus (HIV) infection. The factors affecting adherence to ART, which have not yet been explored in Oman for children living with HIV infection, must thus be investigated.

Methods

A cross-sectional study was conducted that included all children living with HIV who were receiving care at the pediatric infectious disease clinics of Royal Hospital, a tertiary care center, and who had been on pediatric antiretroviral therapy (ART) formulations for more than one year. After receiving informed consent, 43 caregivers of HIV-positive children were interviewed using a questionnaire. Patients’ characteristics, socioeconomic, pharmacological, and health care system factors were analyzed in correlation with adherence to ART using SPSS version 26 (IBM Corp., Armonk, USA).

Results

Thirty percent of patients (n=13) had high HIV viral loads, and among those, 19% (n=8) had suboptimal adherence. Young and non-educated caregivers, low income, and lack of transportation were significant factors for poor adherence to ART therapy, with a significant p-value. Other important factors noticed were medication not available at home, missed medication refills, and a child's busy schedule at school, and patients' caregivers did not always administer the prescribed medications.

Conclusion

The study results support the recommendation to provide a pediatric formulation of ART and supply it to peripheral secondary care hospitals for easy access to medications. Psychological assessments and counselling at the adolescent medicine clinic in the same centre would aid caretakers psychologically and gauge children with HIV's preparedness for disease disclosure.

## Introduction

Between 1984 and 2018, there were 3,060 Omanis diagnosed with human immunodeficiency virus (HIV). A hundred and twenty of them were between the ages of five and 14, and 77 were under the age of four years. The proportion of patients who were under 14 years old at the time of diagnosis decreased dramatically from 14.2% in the period 1984 to 1996 to 2.9% in the period from 2013 to 2018. The former was attributed to the increase in antiretroviral therapy (ART) coverage and other factors [1]. In 2020, Al Sawai et al. estimated the cumulative yearly incidence of HIV infection per million under-14-year-old children in Oman since 1992 as 5.7 per million, and the highest incidence was in 2010 with 11.6 per million, which was secondary to starting national HIV testing for pregnant women as part of the antenatal care program in 2009. It was also found that the children infected with HIV after 2009 were more likely to be asymptomatic and had better clinical outcomes due to early initiation of antiretroviral infection [2]. Currently, 31 children are living with HIV in Oman, being followed by the pediatric infectious disease team at the Royal Hospital [3]. ART significantly improves the clinical, virological, and immunological outcomes for HIV-positive children [4]. Maintaining high ART adherence (>90%) is crucial for successful viral suppression [5-6]. Several studies tried to assess adherence using different methods, but each one resulted in different results with limitations and reflected specific factors in each country [7-9]. Viral load is one of the most important indicators of adherence to antiretroviral therapy that can be done routinely in the outpatient visit. It can also be used as a tool to monitor the disease's progression and to counsel the child and caregiver on the importance of adhering to antiretroviral medications. Undetectable viral load should be the aim for every patient with HIV infection to maintain good health and prevent disease progression, and HIV related opportunistic infections [10]. There is limited local data on factors affecting the compliance with antiretroviral therapy in children living with HIV in the country. The goal of this study was to examine the different factors affecting antiretroviral medication adherence among Omani children living with HIV who were treated at the Royal Hospital from August 2021 to July 2022.

## Materials and methods

Study population

A cross-sectional study was conducted using a questionnaire-based survey answered by the caregivers of children living with HIV. International data that studied adherence to antiretroviral therapy was reviewed, and the questionnaire was developed taking into consideration general factors and local specific factors [5,11]. The survey was initially piloted on 10 families who were followed in the HIV outpatient clinic, and was refined based on their feedback and the clarity of the questions. The survey was written in English and Arabic (Appendices 1-2). All pediatric HIV-positive patients under the age of 13 years who received care and were followed at the pediatric infectious disease clinic at Royal Hospital, Muscat, Oman, were included in the study. Patients who were on an ART regimen for more than a year from August 2021 to July 2022 were eligible to participate in the study. Patients with an undetectable viral load, compliant with the medications, and who had regular medication refills, were considered optimally adherent to ART. Patients who had high viral load, were non-compliant to the medication, due to various factors, and not regularly taking the refill prescription, were considered non-adherent.

Ethical consideration

Ethical approval for this research was obtained from the scientific research committee at the Royal Hospital (SRC#111/2021). Informed consent was obtained from the caregivers of children included in the study (Appendix 3).

Data collection

The research team used a questionnaire to interview caregivers. Data on the child's and the caregiver's demographics, including age, gender, educational attainment, place of residence, the existence of other comorbid conditions, parents' HIV status, and disclosure of HIV condition to the child and the family, were gathered by research investigators. It also explored different factors influencing adherence to ART, like factors related to the child, caregiver, socioeconomic status, pharmacological, and health care system-related factors. Other medical data, such as viral load and lymphocyte subset count status, were obtained from the Al Shifa electronic health care system.

Data analysis

Data were analyzed using SPSS version 26 (IBM Corp., Armonk, USA). Frequency tables were used for categorical variables. Chi-square test was used to show the association between suboptimal adherence and high viral load and low lymphocyte subset (CD4 count <500). Fisher’s exact test was used to show the significance of different factors influencing adherence to ART and suboptimal adherence in patients with high viral load. Factors that showed a p-value <0.05 were considered statistically significant.

## Results

Patient demographics

**Figure 1:**
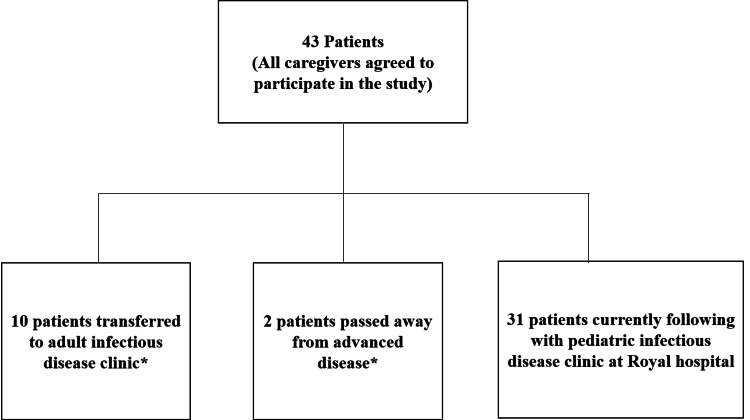
Flow Chart of The Population Included in The Study from August 2021 to July 2022 * After the study period

This study included patients who were on ART for more than a year from August 2021 to July 2022. During the study period they were 43 children living with HIV being followed in the pediatric HIV clinic at the Royal Hospital until July 2022. By the end of the year 2022, 10 adolescents above the age of 13 years transferred to the adult services with initial joint clinics between pediatric and adult HIV services, and two patients passed away with advanced disease (Figure 1).

Children over the age of 10 years made up the majority (51.1%), followed by the age group between five and 10 years (37.2%), and patients under the age of five years (11.6%). There were 51% female patients and 49% male patients. Biological parents made up 88% of the caretakers, with the remainder being other relatives. Almost 91% of the caregivers were female, and the remaining are male. Sixty percent of caregivers were between the ages of 30 and 45 years, 23% were over 45 years, and 16% were less than 35 years of age.

Factors influencing adherence to ART

**Figure 2:**
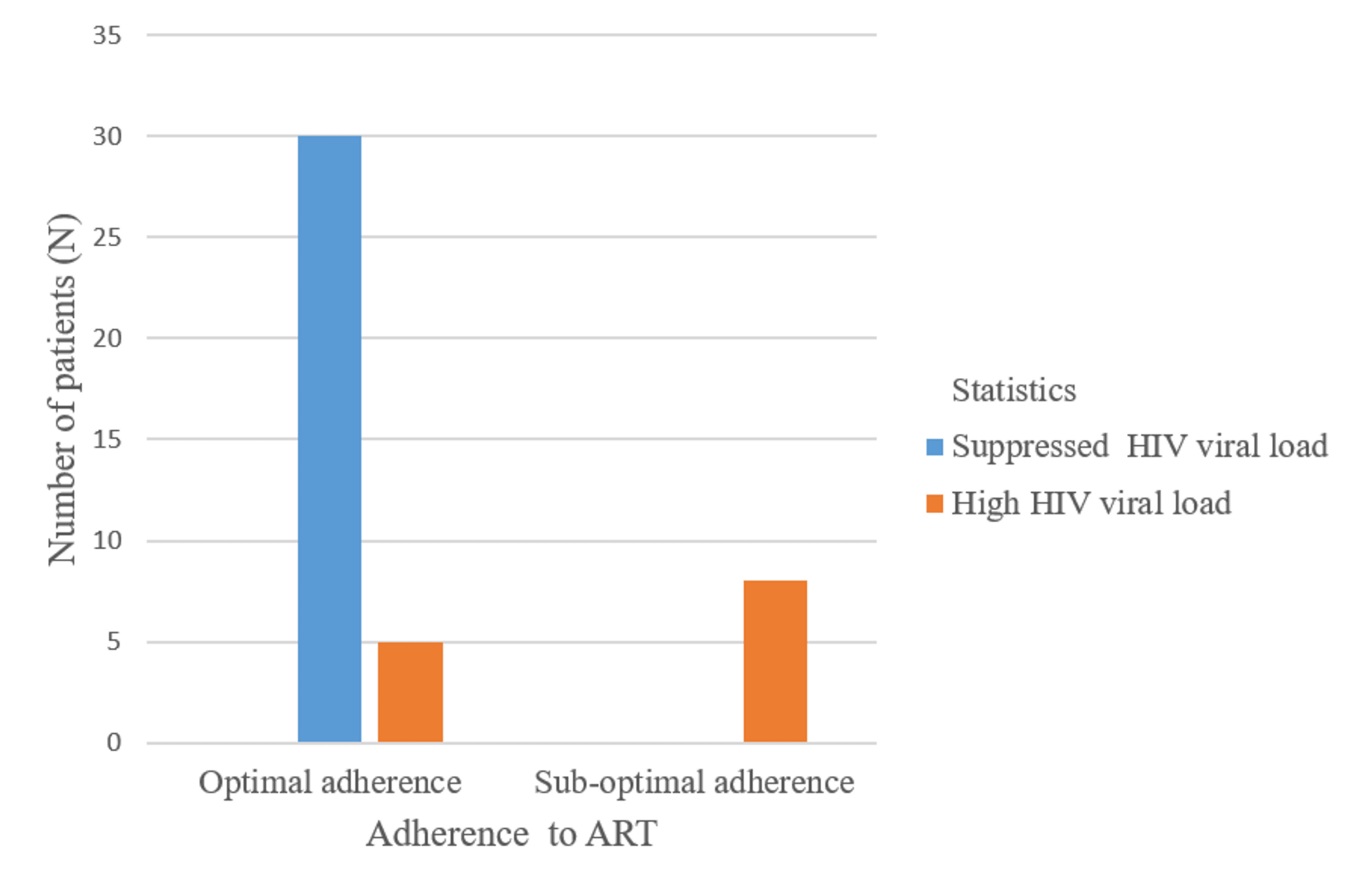
Correlation Between Adherence to ART and HIV Viral Load ART: antiretroviral therapy; HIV: human immunodeficiency virus.

About 30% of patients had elevated viral loads as a result of various reasons. Low lymphocyte subset (CD4 count <500) was present in 18.1% of patients. About 19% of patients' caregivers reported non-compliance with ART (not taking the medications regularly as prescribed). Optimal adherence was defined as taking more than 95% of the prescribed doses per month, and suboptimal adherence was defined as taking less than 95% of the prescribed doses per month. Our study showed that 30 patients who are optimally adherent to ART had suppressed HIV viral load (Figure 2).

Patient Related Factors

Of the 23% of children who knew about the illness, all were older than 10 years (Table 1).

**Table 1:** Patient and Caregiver Demographics, Categorized to Optimal and Suboptimal Adherence ART: antiretroviral therapy.

		Adherence		
				Patient		Optimal adherence	Sub-Optimal adherence	p- value
						N (%)	N (%)	
Age	< 5 years	4 (9.3)	1 (2.3)	0.672
	5-10 years	12 (27.9)	4 (9.3)	
	> 10 years	19 (44.1)	3 (6.9)	
Gender	Male	19 (44.1)	2 (4.6)	0.24
	Female	16 (37.2)	6 (13.9)	
Enrolled in school	Yes	29 (67.4)	5 (11.6)	0.332
	No	6 (13.9)	3 (6.9)	
Duration of ART	< 3 years	11 (25.5)	4 (9.3)	0.358
	3 – 5 years	3 (6.9)	1 (2.3)	
	> 5years	21 (48.8)	3 (6.9)	
Medications are given to the child by	Child himself	11 (25.5)	2 (4.6)	0.356
	Parents	20 (46.5)	6 (13.9)	
	Caregiver- relative	4 (9.3)	0 (0)	
The child is aware of the disease	Yes	10 (23.2)	0 (0)	0.165
	No	25 (58.1)	8 (18.6)	
The child is aware of the relationship between non-adherence and advanced disease	Yes	23 (53.4)	3 (6.9)	0.23
Caregivers				
Age	< 30 years	3 (6.9)	4 (9.3)	0.009
	31-45 years	22 (51.1)	4 (9.3)	
	> 45 years	10 (23.2)	0 (0)	
Gender	Male	4 (9.3)	0 (0)	1
	Female	31 (72.0)	8 (18.6)	
Caregivers are	Parents	30 (69.7)	8 (18.6)	0.565
	Relatives	5 (11.6)	0 (0)	
Education level	None	3 (6.9)	4 (9.3)	0.022
	Primary	9 (20.9)	1 (2.3)	
	Secondary	19 (44.1)	1 (2.3)	
	Tertiary	4 (9.3)	2 (4.6)	
Co-morbidity	Yes	25 (58.1)	5 (11.6)	0.681
	No	10 (23.2)	3 (6.9)	
Awareness about the disease	Yes	33 (76.7)	8 (18.6)	1
	No	2 (4.6)	0 (0)	

Medications were not given by the caregivers to their children because 60% of patients (p-value 0.037) did not feel sick. Despite the fact that 55.8% of patients were on ART for longer than five years, patient adherence to ART was not affected. Though children who were self-medicating presented 30% of our study population, it didn’t show any significant correlation to suboptimal adherence. Despite that, 60% of children knew the relationship between suboptimal adherence and advanced disease (though not all of them knew about the disease), but it didn’t show a significant impact on compliance to ART. 

Caregiver Related Factors

The caregiver’s age showed a significant influence on adherence of children to ART. About 57.1% of patients with inadequate adherence had caregivers who were younger than 30 and between 31 and 45 years old (p-value 0.009) (Table 1). Our results showed that non-educated caregivers showed suboptimal adherence in 57.1% (p-value 0.022). Around 69.8% of caregivers had other medical co-morbid conditions, but it was not a significant factor affecting medication adherence. Two caregivers (4.7%) were not aware of the child’s disease, though it didn’t show an impact on adherence to ART.

Socio-Economic Related Factors

Around 11.4% of children were not living with parents, and extended families counted for 44.2% (Table 2). Though 90% of caregivers disclosed their HIV status to family members, 79.1% had family support according to the survey. Out of 79.1% of children who were enrolled in school; 44.2% had school support, especially for outpatient department (OPD) visit appointments. ART medications were not given to the child due to the child's presence at school during the timing of the medication. Therefore, it led to suboptimal adherence, which was reported in 80% of patients (p-value 0.003). With a p-value of 0.049, low income [monthly salary <400 Omani Rial (OMR)] was strongly linked to inadequate adherence [12]. Lack of transportation, due to low income, led to missed medication refills. This was determined by the dispensary of the medication for a refill from the pharmacy. It showed a significant relation with suboptimal adherence (p-value 0.031) and detectable viral load.

**Table 2:** Socioeconomic Factors Influencing Adherence to ART HIV: human immunodeficiency virus; ART: antiretroviral therapy.

		Adherence		
				Socioeconomic factors		Optimal adherence	Sub-Optimal adherence	p- value
						N (%)	N (%)	
The child is living with parents	Yes	32 (74.4)	6 (13.9)	0.228
	No	3 (6.9)	2 (4.6)	
Family	Extended	15 (34.8)	4 (9.3)	1
	Nuclear	20 (46.5)	4 (9.3)	
Income	Low	10 (23.2)	2 (4.6)	0.049
	Average	24 (55.8)	6 (13.9)	
	High	1 (2.3)	0 (0)	
Disclosure of HIV status	Yes	31 (72.0)	8 (18.6)	1
	No	4 (9.3)	0 (0)	
Family support	Yes	26 (60.4)	8 (18.6)	0.171
	No	9 (20.9)	0 (0)	
School support	Yes	16 (37.2)	3 (6.9)	0.789
	No	2 (4.6)	1 (2.3)	
	Not applicable	17 (39.5)	4 (9.3)	
	(not enrolled in school)			

Medication-Related Factors

Medications were not given to the child, as self-reported by the caregivers, due to various reasons (Table 3).

**Table 3:** Medication Related Factors Influencing Adherence to ART ART: antiretroviral therapy.

		Adherence		
				Medications are not given due to		Optimal adherence	Sub-Optimal adherence	p- value
						N (%)	N (%)	
Forgetfulness	Yes	5 (11.6)	1 (2.3)	1
	No	30 (69.7)	7 (16.2)	
Drug finished	Yes	5 (11.6)	5 (11.6)	0.01
	No	30 (69.7)	3 (6.9)	
Caregiver illness	Yes	3 (6.9)	1 (2.3)	0.228
	No	32 (74.4)	7 (16.2)	
Busy work schedule	Yes	3 (6.9)	1 (2.3)	0.576
	No	32(74.4)	7 (16.2)	
Family problems	Yes	4 (9.3)	2 (4.6)	0.308
	No	31 (72.0)	6 (13.9)	
Child's school schedule	Yes	1 (2.3)	4 (9.3)	0.003
	No	34 (79.0)	4 (9.3)	
Illness of child	Yes	2 (4.6)	0 (0)	1
	No	33 (76.7)	8 (18.6)	
Lack of trust in the efficacy of the drug	Yes	2 (4.6)	2 (4.6)	0.151
	No	33 (76.7)	6 (13.9)	
Bad drug taste	Yes	17 (39.5)	0 (0)	0.014
	No	18 (41.8)	8 (18.6)	
Child vomiting the medications	Yes	14 (32.5)	2 (4.6)	0.688
	No	21 (48.8)	6 (13.9)	
Child refused drugs	Yes	13 (30.2)	4 (9.3)	0.692
	No	22 (51.1)	4 (9.3)	
Side effects	Yes	3 (6.9)	0 (0)	1
	No	32 (74.4)	8 (18.6)	
The child was not feeling sick	Yes	2 (4.6)	3 (6.9)	0.037
	No	33 (76.7)	5 (11.6)	
Caregivers give herbal medicine	Yes	2 (4.6)	0 (0)	1
	No	33 (76.7)	8 (18.6)	
Missed medication refill due to the availability of the drug at home	Yes	14 (32.5)	8 (18.6)	0.004
	No	21 (48.8)	0 (0)	

Out of different factors, 39.5% of patients’ caregivers reported bad drug taste, and children refusing the medications. Vomiting the medications was reported in 37.2% of the children. Despite this, vomiting was not associated with suboptimal adherence. Caregivers missed a refill of medication prescription due to available previous medication stock at home, caregivers didn't dispense their monthly doses from the pharmacy, and self-reported that they have available medication stock at home, which was related to suboptimal adherence with a p-value of 0.004. Forgetfulness, illness of caregivers, family problems, child school schedule, illness of child/caregiver, presence of comorbidities, lack of trust in efficacy of the drug, drug side effects, and using herbal medications did not contribute significantly to suboptimal adherence.

Pharmacological-Related Factors

The results showed that 81.4% of caregivers knew when to repeat the dose if the child spilled the medication and also knew how to manipulate the dose using a syringe or pouring the liquid into a cup. Due to a shortage of some pharmaceutical formulations, 20.9% of patients substituted another formulation. About 60.5% of patients had changes in medication formulation from syrup to tablet or from combination pill to individual component to improve their compliance. Concerning the various prescription brands they obtained from the pharmacy, 25.6% of caregivers expressed confusion, related to the type of medication or change in the formulation due to a change in medication brands, depending on the medical supply. Around 41.9% of those who provided care reported that it was challenging to give the child their medications twice per day. Around 27.9% developed resistance to medication. None of the pharmacological factors showed an impact on adherence to ART (Table 4).

**Table 4:** Pharmacological Factors Influencing Adherence to ART ART: antiretroviral therapy.

		Adherence		
				Pharmacological factors		Optimal adherence	Sub-Optimal adherence	p- value
						N (%)	N (%)	
Can easily measure the syrup dose	Yes	31 (72.0)	5 (11.6)	0.106
	No	4 (9.3)	3 (6.9)	
Knows when to repeat the dose if the child spills the medication	Yes	30 (69.7)	5 (11.6)	0.153
	No	5 (11.6)	3 (6.9)	
Knows how to manipulate the dose using a syringe or pour the liquid into a cup	Yes	30 (69.7)	5 (11.6)	0.153
	No	5 (11.6)	3 (6.9)	
Experience swallowing difficulties	Yes	7 (16.2)	3 (6.9)	0.362
	No	28 (65.1)	5 (11.6)	
Able to differentiate fridge items from medication	Yes	35 (81.3)	8 (18.6)	-
	No	0 (0)	0 (0)	
Need to change medication regimen due to out-of-stock formulation	Yes	9 (20.9)	0 (0)	0.171
	No	26 (60.4)	8 (18.6)	
Changed formula from syrup to tablet or from combination pill to individual component	Yes	23 (53.4)	3 (6.9)	0.23
	No	12 (27.9)	5 (11.6)	
Confused about the different medication brands received from the pharmacy	Yes	11 (25.5)	0 (0)	0.09
	No	24 (55.8)	8 (18.6)	
Difficulty in taking the medication twice daily	Yes	14 (32.5)	4 (9.3)	0.701
	No	21 (48.8)	4 (9.3)	

Healthcare System-Related Factors

Almost all caregivers gave positive feedback about the infectious diseases team and were satisfied with the healthcare system and service provided by the team at the Royal Hospital. Thus, it doesn’t show an impact on adherence to ART (Table 5).

**Table 5:** Healthcare System-Related Factors

		Adherence		
				Healthcare system-related factors		Optimal adherence	Sub-Optimal adherence	p- value
						N (%)	N (%)	
Service is acceptable	Yes	35 (81.3)	8 (18.6)	-
	No	0 (0)	0 (0)	
Long waiting list for follow-up	Yes	7 (16.2)	0 (0)	0.315
	No	28 (65.1)	8 (18.6)	
Impatient or unsympathetic staff	Yes	0 (0)	1 (2.3)	0.186
	No	35(81.3)	7 (16.2)	
Enough time for consultation/counseling	Yes	35 (81.3)	8 (18.6)	-
	No	0 (0)	0 (0)	
Easy to reschedule missed appointment	Yes	35 (81.3)	8 (18.6)	-
	No	0 (0)	0 (0)	

All caregivers reported that they had enough time for consultation and counseling. About 83.7% of caregivers reported they have a convenient clinic appointment waiting list. It was easy to reschedule missed appointments for all patients (100%). Causes of missing clinic appointments were evaluated (Table 6).

**Table 6:** Causes of Missing Clinic Appointments

		Adherence		
				Missed clinic appointment due to		Optimal adherence	Sub-Optimal adherence	p- value
						N (%)	N (%)	
Forgetfulness	Yes	7 (16.2)	0 (0)	0.315
	No	28 (65.1)	8 (18.6)	
Still has medications at home	Yes	19 (44.1)	4 (9.3)	1
	No	16 (37.2)	4 (9.3)	
Unable to get permission from the workplace	Yes	10 (23.2)	2 (4.6)	1
	No	25 (58.1)	6 (13.9)	
Illness of the caregiver	Yes	6 (13.9)	3 (6.9)	0.332
	No	29 (67.4)	5 (11.6)	
Family problems	Yes	6 (13.9)	2 (4.6)	0.629
	No	29 (67.4)	6 (13.9)	
Transportation not available	Yes	11 (25.5)	6 (13.9)	0.042
	No	24 (55.8)	2 (4.6)	
Child at school	Yes	13 (30.2)	1 (2.3)	0.24
	No	22 (51.1)	7 (16.2)	

Children’s caregivers missed a clinic appointment mainly because they were still taking medications at home (53.5%), due to a lack of transportation (39.5%), and the child's school schedule (32.6%). 

## Discussion

ART showed a major effect in the long-term survival of children living with HIV [13]. Poor adherence to ART could lead to treatment failure, its associated morbidity and mortality, and development of drug resistance [11,14]. A Brazilian study showed that optimal adherence to ART is the main factor that contributes to virological suppression despite using genotypic tests to guide the choice of ART regimen [15]. This study showed virological failure in 61.5% of patients with suboptimal adherence compared to 38.5% of patients with optimal adherence. A study that was conducted in India on a larger study population showed almost similar virological failure percentage in both groups (26.7% in the suboptimal adherence group and 15.3% in the optimal adherence group) [5]. Children living with HIV require caregiver and family support to maintain good compliance with medications [11]. Patient-related factors, including age and gender, showed no impact on adherence to ART, which is similar to reports from other studies [5]. HIV disclosure to the patient showed a significant impact on adherence. Our study showed that patients are not feeling sick; hence not taking their medications. It showed significant non-adherence to antiretroviral therapy in 60% (p-value 0.037).

In our study population, 23% of patients aged from 10 to 12 years know their HIV status. This result is similar to the disclosure status in a study conducted in Ghana, which showed HIV disclosure status of 21% of children living with HIV aged from eight to 14 years [11]. Children who were not aware of their HIV status showed poor adherence to ART in the same study [11]. Another Indian study also showed that children with full HIV status disclosure never reported non-compliance to medications [5]. On the other hand, studies conducted in high-income countries showed that HIV disclosure to the patient was associated with poor adherence [16-17]. The role of caregivers in administering antiretroviral medications to their children is crucial, and their psychological support remains important while the child becomes older and independent in taking the medications. Thus, caregivers’ educational level is important in compliance with ART [5].

Our study showed that younger caregivers' age is associated with less adherence to ART. It also showed that non-educated caregivers are a significant factor leading to suboptimal adherence. A similar result was obtained from a Ghanaian study, which showed that caregivers with a higher educational level were associated with optimal adherence [11]. Our results showed that low income is a significant factor related to suboptimal adherence. It was reported in one study that adherence reached 50-80 % in low-income settings [18]. Unavailability of medications and refusal to take the medications are significant factors leading to virological failure in the Indian study [5]. Our study showed that pharmacological factors, including unpalatable drug taste and refusal to take the medications, were not significantly related to suboptimal adherence. Although some children were provided with different medication formulations, as some of the medications were out of stock during the study period, it was not related to poor adherence. Our participants showed good satisfaction with the health care system and service provided to the patients. Caregivers reported that they missed some clinic days due to a lack of transportation and the child's school schedule. Despite this, it was not related to suboptimal adherence. On the other hand, an Indian study showed that inappropriate clinic timing and lack of transportation are potential factors that influence adherence [5]. Our study has some limitations, which include a small sample size, recall bias of the caregiver, and caregiver interpretation of the questions included in the survey.

## Conclusions

In conclusion, adherence to ART in children living with HIV is influenced by various factors. Children unaware of HIV status, school schedule, caregiver’s age and education level, low income, lack of transportation, and missing medication refill are leading factors to suboptimal adherence in this study. These factors have to be addressed to improve the care of children living with HIV. Providing pediatric formulation of ART and supplying it to peripheral secondary care hospitals for easy access to medications is also an important recommendation obtained from this survey. Psychological assessments and counselling at the adolescent medicine clinic in the same centre are important factors in caring for children with HIV infection and would aid caregivers psychologically and gauge children with HIV's preparedness for disease disclosure.

## Figures and Tables

**Figure 3:**
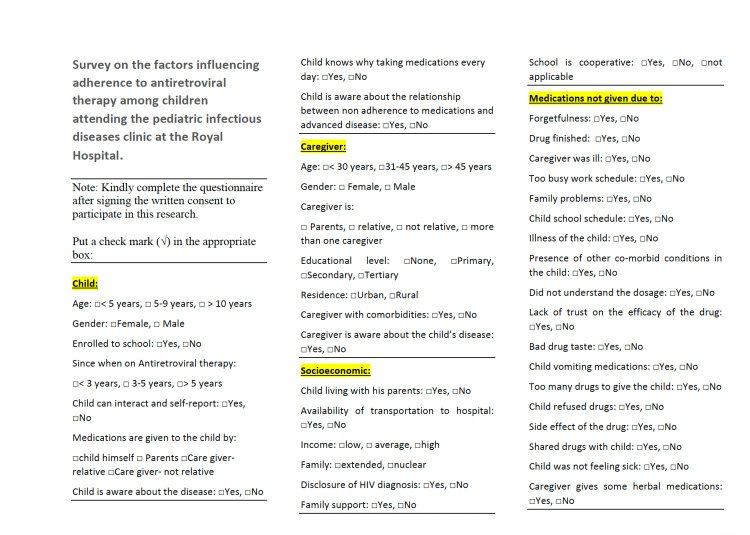
English Questionnaire - Part 1

**Figure 4:**
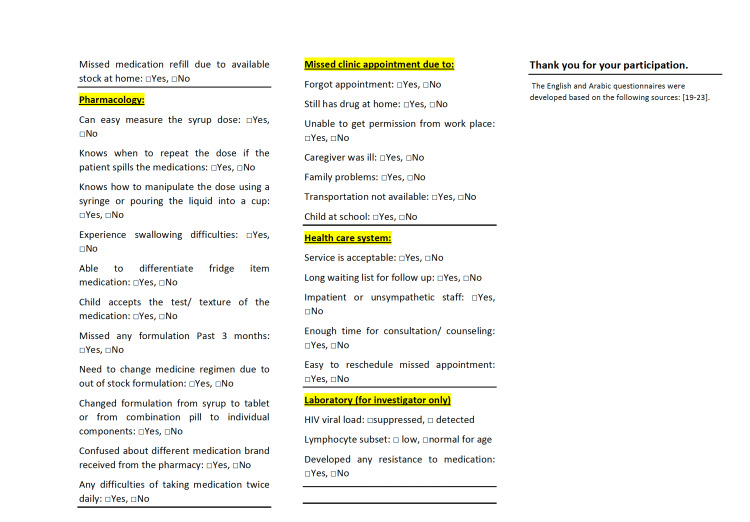
English Questionnaire - Part 2

**Figure 5:**
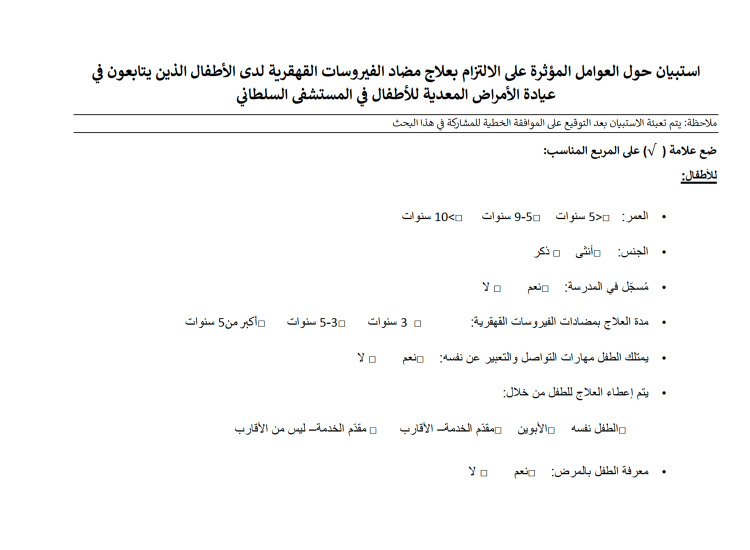
Arabic Questionnaire - Part 1

**Figure 6:**
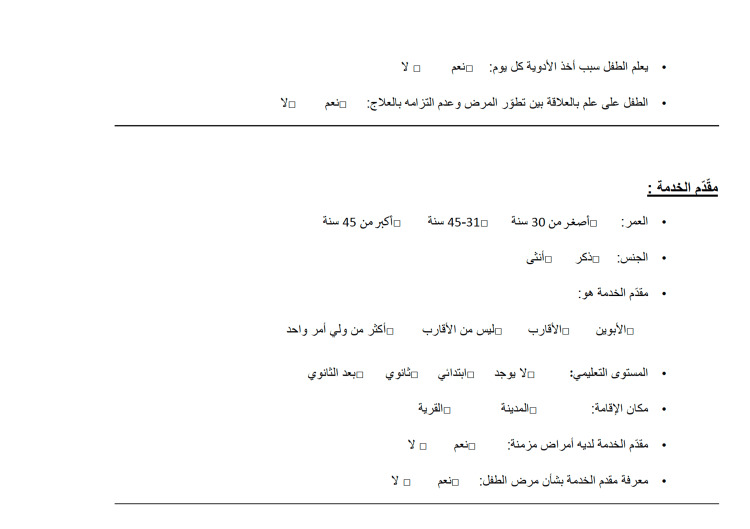
Arabic Questionnaire - Part 2

**Figure 7:**
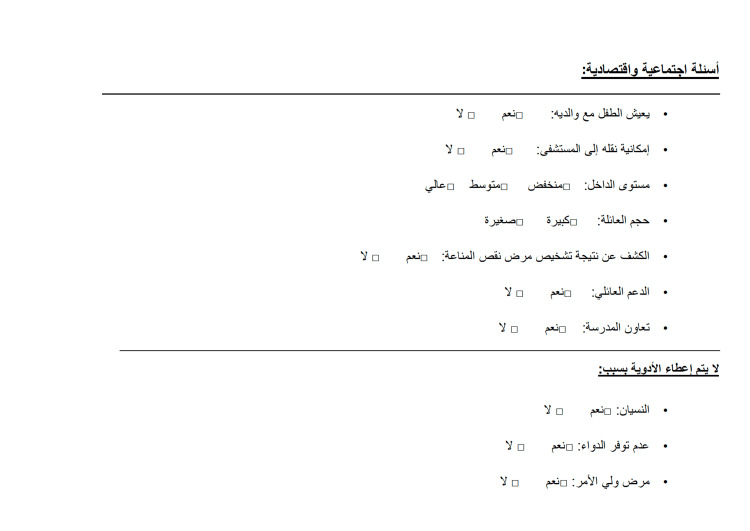
Arabic Questionnaire - Part 3

**Figure 8:**
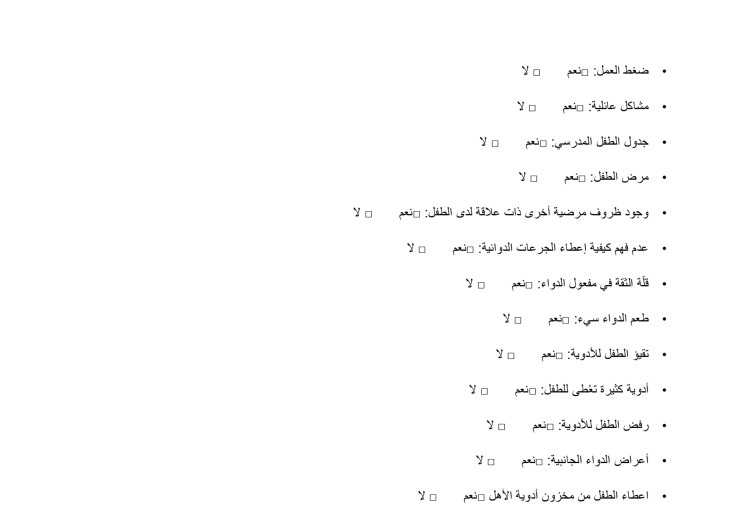
Arabic Questionnaire - Part 4

**Figure 9:**
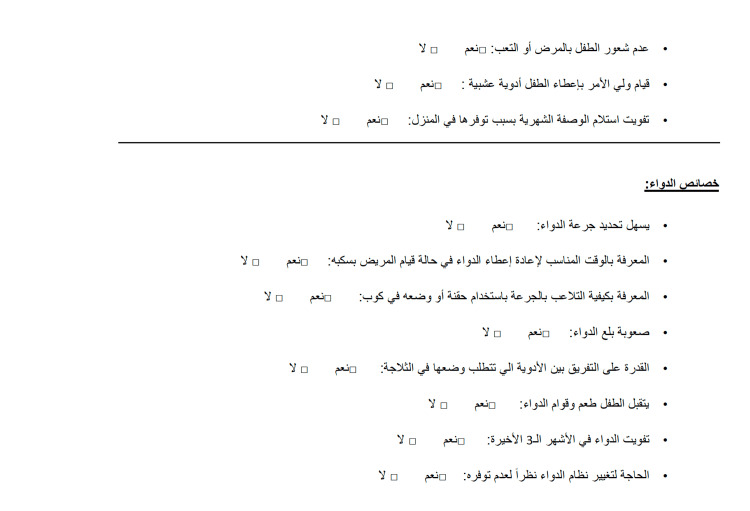
Arabic Questionnaire - Part 5

**Figure 10:**
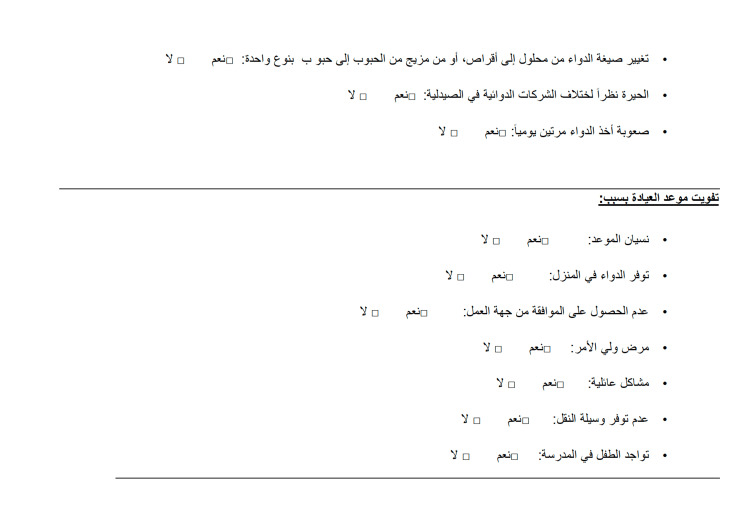
Arabic Questionnaire - Part 6

**Figure 11:**
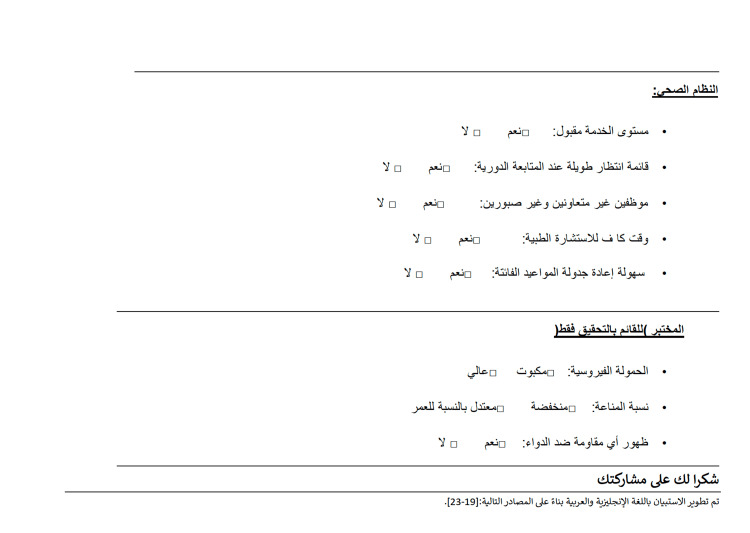
Arabic Questionnaire - Part 7

## Appendices

Appendix 2

English Questionnaire 

Appendix 3

Arabic Questionnaire

Appendix 3

Consent Form

Title of the study: Factors influencing adherence to pediatric antiretroviral therapy at Royal Hospital, Oman

Supervisor: Dr Hilal Al Hashami, Pediatric Infectious Disease Consultant at Royal Hospital. Email: hashamihs@gmail.com

Principal investigator: Zulfa Khalfan Al Shibli, Pediatric resident. Email: Zulfa.s19@resident.omsb.org

Co-investigators: Basma AL Jabri, Clinical Pharmacist. Email: Phbasmaa1@gmail.com and Jalila AL Namaani, Infectious Diseases Nurse. Email: Jkmomania@gmail.com

Purpose of the study: Our aim is to study the factors that are affecting adherence to Human immunodeficiency virus (HIV) therapy among Omani children being followed at the Royal Hospital. We will conduct this study over 1 year. We will interview the participants using a questionnaire. Factors from different aspects will be analyzed to see if they are influencing adherence to HIV therapy. Participants will be followed up with once or twice a year only.
 

Benefits: We are conducting this study to provide recommendations for optimal adherence to HIV therapy to achieve a better long-term outcome for children with HIV.

Confidentiality: Participants’ privacy and confidentiality will be maintained throughout the research process. We will assign a number for each participant that will be used during documentation and data analysis. The researchers will make sure that documents will be kept in safe hands and will be accessed only by the research investigators. For any further queries, you can contact the researcher at the email address mentioned above.

Voluntary participation: Participation in this research is voluntary. If you agree to be part of this study kindly sign on this consent form below. You can withdraw at any point in time without giving reasons.

CONSENT

·  I have read all the information mentioned above, and I understand that my participation is voluntary. I can withdraw at any time.

·  I voluntarily agree to participative/, or my child participates in this study. Participant's /or caregiver signature ____________ Date __________ Investigator's signature _________________ Date __________

--------------------------------------------------------------------------------------------------------------------------------
